# Serum CD166: A novel biomarker for predicting nasopharyngeal carcinoma response to radiotherapy

**DOI:** 10.18632/oncotarget.16399

**Published:** 2017-03-21

**Authors:** Huan Lin, Ze-Tan Chen, Xiao-Dong Zhu, Ling Li, Song Qu, Zhao Wei, Fang Su, Jing-Ni Wei, Zhong-Guo Liang, Qi-Yan Mo, Jiang-Bo Wu, Hui-Ling Meng

**Affiliations:** ^1^ Department of Radiation Oncology, Affiliated Cancer Hospital of Guangxi Medical University and Cancer Institute of Guangxi Zhuang Autonomous Region, Nanning, Guangxi 530021, P.R. China; ^2^ Guangxi Key Laboratory of Early Prevention and Treatment for Regional High Frequency Tumor, Guangxi Medical University, Nanning, Guangxi 530021, P.R. China; ^3^ Key Laboratory of High-Incidence-Tumor Prevention and Treatment (Guangxi Medical University), Ministry of Education, Nanning, Guangxi 530021, P.R. China

**Keywords:** nasopharyngeal carcinoma, radiosensitivity, radioresistance, biomarker, CD166/ALCAM

## Abstract

The present study aimed to identify whether CD166 can be used as a biomarker for predicting the response of nasopharyngeal carcinoma (NPC) to radiotherapy. The serum concentration of CD166 in patients with NPC were detected by enzyme-linked immunosorbent assay. The secreted level of CD166 with radioresistant NPC was significantly higher than that with radiosensitive NPC. *In vitro*, the CD166 positive rate in the CNE2 cell membrane was significantly lower than that in the CNE2R cell membrane. The magnetic-activated cell sorting technology was used to obtain CNE-2R-CD166(+) and CNE-2R-CD166(−) cell lines. Then radiosensitivity, cell proliferation, and apoptosis were assessed using colony formation assay, cell counting kit 8 assay (CCK-8), and flow cytometry, respectively. The radiation sensitivity ratio was 1.28, indicating that the CNE2R-CD166(−) cells had a stronger radiation sensitivity. The result of CCK-8 assay indicated that the survival fraction of CNE2R-CD166(+) cells was significantly higher than that of CNE2R-CD166(−) cells. The apoptotic rate of CNE2R-CD166(+) cells was significantly lower than that of CNE2R-CD166(−) cells. Our data demonstrate that the secreted protein CD166 may be can used as a biomarker for predicting the response of NPC to radiotherapy.

## INTRODUCTION

Nasopharyngeal carcinoma (NPC) is one of the most common malignant tumors in Southeast Asia and southern China [1, 2]. Because of its special anatomical position and the sensitivity to radiation, the radiation therapy is the main treatment method [3]. The local recurrence and distant metastasis of NPC in patients after radiotherapy is a bottleneck to restrict the curative effect and prognosis, and the radiation resistance is one of the main reasons [4, 5]. Therefore, it is of great significance to search for molecular markers for predicting the sensitivity of NPC radiation therapy and for guiding the treatment of NPC and improve its prognosis.

The occurrence of radiation resistance is a multistage process, with multiple genes involved. The change in its gene level inevitably leads to changes in a large number of secretory proteins. Identification of differentially expressed proteins by proteomic methods is an effective and widely used technique [6]. In previous studies [7], the radioresistant CNE-2R and its parental CNE-2 cell line were cultured *in vitro*. Some differentially secreted proteins were identified by proteomic techniques, and CD166 is one of them. The abnormal expression of CD166 according to Western blot analyses may serve as a potential biomarker for predicting NPC response to radiotherapy.

The levels of secreted protein CD166 were detected in the serum of patients with NPC in this study to understand the relationship between radiosensitivity and clinical parameters. NPC cell line CNE2R and its parental cell line CNE2 were cultured *in vitro*, and the cell lines CNE-2R-CD166(+) and CNE-2R-CD166(−) were obtained by the magnetic-activated cell sorting (MACS) technology. Then, radiosensitivity, cell proliferation, and apoptosis were assessed using colony formation assay, cell counting kit 8 (CCK-8) assay, and flow cytometry, respectively. The study aimed to investigate the relationship between the expression of secreted protein CD166/activated leukocyte cell adhesion molecule (ALCAM) and the sensitivity of NPC radiotherapy, and to identify whether it could be used as a biomarker for predicting the response of NPC to radiotherapy.

## RESULTS

### Concentration of secreted protein CD166 was detected by ELISA

**Figure 1:**
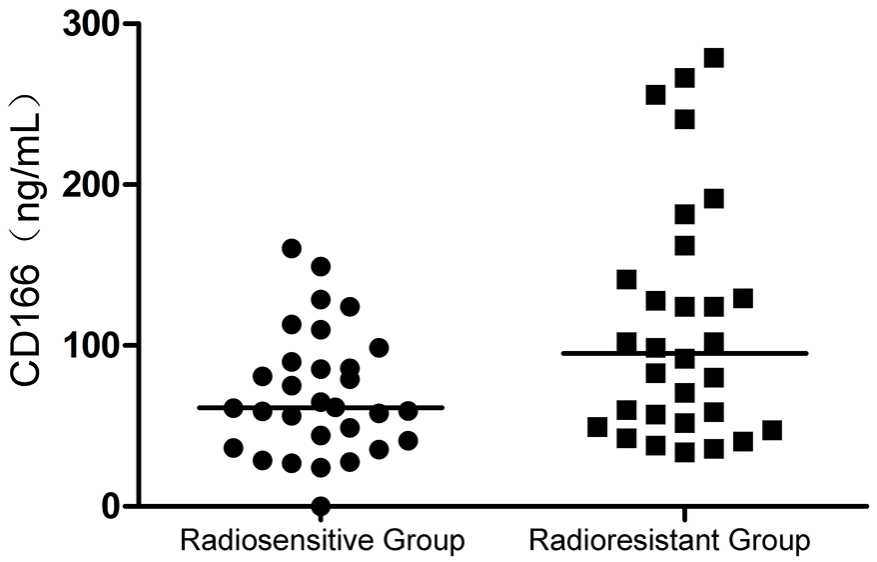
Concentration of secreted protein CD166 was higher in the radioresistant group than that in the radiosensitive group; the difference had statistical significance (P = 0.03)

ELISA showed that the expression of secreted protein CD166 in the serum of patients in the two groups had a significant difference (*P* = 0.03), as shown in Figure 1. The concentration of secreted protein CD166 in the radioresistant group was higher than that in the radiosensitive group. No correlation was observed between CD166 expression and clinical stage, T stage, N stage, and other clinical parameters, include pathological type.

### Positive rate of CD166 was higher in the CNE2R cell membrane than in the CNE2 cell membrane

**Figure 2:**
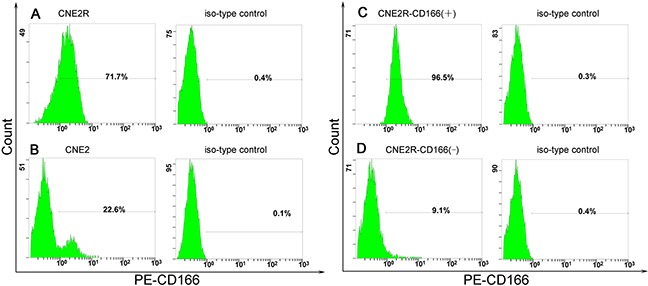
**(A) Positive rates of CD166 in the CNE2R cell membrane was (69.13 ± 5.15)%. (B)** Positive rates of CD166 in the CNE2 cell membrane was (21.37 ± 1.50)%. *The positive rate of CD166 at CNE2R was significantly higher than the positive rates of CD166 at CNE2 (P < 0.05). **(C)** Positive rates of CD166 at CNE2R-CD166(+) cells was 96.5% after immunomagnetic separation. **(D)** Positive rates of CD166 at CNE2R-CD166(−) was 9.1% after immunomagnetic separation.

The positive rate of CD166 in the CNE2R and CNE2 cell membranes was detected by flow cytometry before immunomagnetic separation. The positive rate of CD166 was significantly lower in the CNE2 cell membrane than in the CNE2R cell membrane [(21.37 ± 1.50)% vs (69.13 ± 5.15)%, *P* < 0.01] (Figure 2A and Figure 2B). CNE2R-CD166(+) and CNE2R-CD166(−) cells were obtained by the MACS technology as mentioned earlier. The positive rate of CD166 at CNE2R-CD166(+) and CNE2R-CD166(−) cells was detected by flow cytometry directly. The positive rate of CD166 at CNE2R-CD166(+) cells and CNE2R-CD166(−) cells were 96.5% and 9.1% respectively (Figure 2C and Figure 2D), indicating that the sorting purity was high.

### Radiation sensitivity of the CNE2R-CD166(+) and CNE2R-CD166(−) cell lines

**Figure 3:**
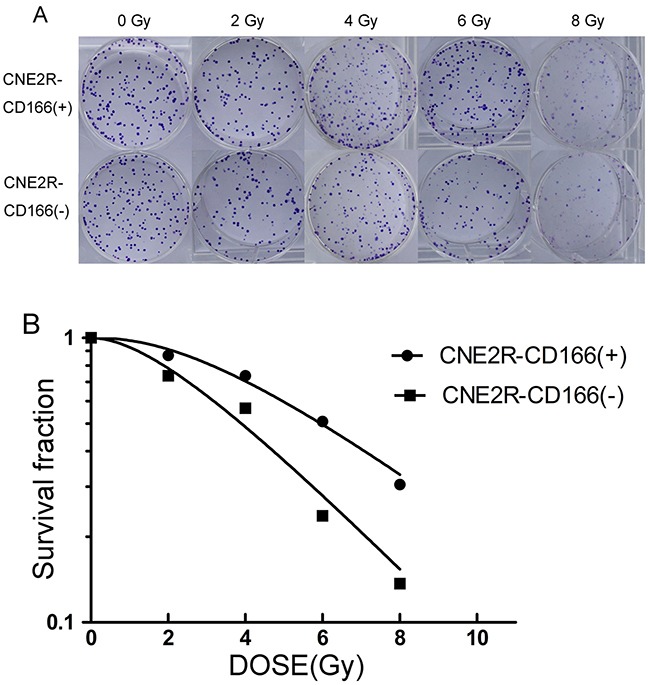
Colony formation assays for radiosensitivity **(A)** Colony formation assays of the CNE2R-CD166(+) and CNE2R-CD166(−) cell lines were performed to evaluate radiosensitivity. **(B)** Fit curves were analyzed using GraphPad Prism 5.0 software.

Survival curves of the CNE2R-CD166(+) and CNE2R-CD166(−) cell lines were plotted using colony formation assays. Colony formation assays of the CNE2R-CD166(+) and CNE2R-CD166(−) cell lines were performed to evaluate radiosensitivity (Figure 3A). Figure 3B shows that the survival curve of CNE2R-CD166(+) cells was higher than that of CNE2R-CD166(−) cells. The main parameters are shown in Table 1. From the D_0_(dose of radiation producing a 37% survival rate), Dq (quasi-threshold dose required for cell damage) and SF_2_ (survival fraction at 2 Gy) values, it can be concluded that CNE2R-CD166(−) cells had a greater decrease in the surviving fractions when compared with CNE2R-CD166(+) cells. When the data were analyzed by the multi-target single-hit model. SF = 1 – (1 – e^−*D*/*D*0^)*^N^*, by calculating the sensitization enhancement ratio (SER), we used the D_0_ of CNE2R-CD166(−) cells divided by the D0 of CNE2R-CD166(+) cells, the values of SER_D0_= 1.28>1, suggesting that CNE2R-CD166(−) cells were more sensitive to radiation compared with CNE2R-CD166(+) cells.

**Table 1:** Correlation parameters in the multitarget single-hit model

Group	SF_2_	D_0_ (Gy)	Dq (Gy)
CNE2R-CD166(+)	0.88	4.39	4.08
CNE2R-CD166(−)	0.80	3.44	2.76

### Cell proliferation after irradiation was examined by the CCK-8 assay

**Figure 4:**
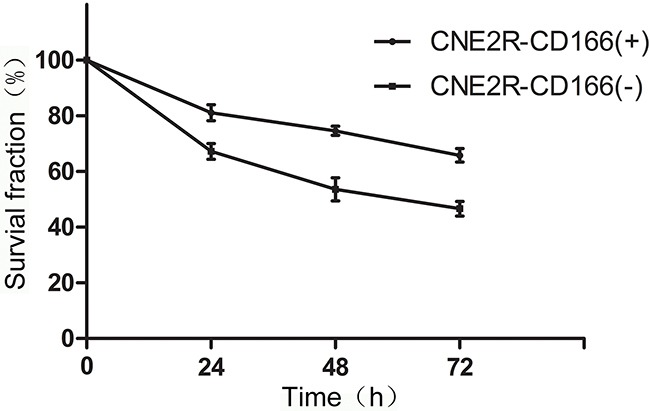
Survival curve after irradiation was detected by the CCK-8 assay in CNE2R-CD166(+) and CNE2R-CD166(−) cells The survival fraction of CNE2R-CD166(+) cells was significantly higher than that of CNE2R-CD166(−) cells at 24, 48, and 72 h.

The comparison of survival fraction between CNE2R-CD166(+) cells and CNE2R-CD166(−) cells at different times after 10 Gy irradiation is shown in Table 2. Figure 4 shows that the survival fraction of CNE2R-CD166(+) cells was significantly higher than that of CNE2R-CD166(−) cells at 24, 48, and 72 h, respectively, indicating that CNE2R-CD166(−) cells were more sensitive to radiation compared with CNE2R-CD166(+) cells.

**Table 2:** Comparison of survival fraction (%) between two groups

Groups	0 h	24 h	48 h	72 h
CNE2R-CD166(+)	100	81.16 ± 2.84	74.64 ± 1.66	65.83 ± 2.41
CNE2R-CD166(−)	100	67.24 ± 2.83	53.60 ± 4.19	46.68 ± 2.63
*P*	—	0.004	0.001	0.001

### Flow cytometry detected the apoptosis of CNE-2R-CD166(+) and CNE-2R-CD166(−) cells

Cell apoptosis was measured by flow cytometry. No significant difference was found between CNE2R-CD166(+) and CNE2R-CD166(−) cells [(4.83 ± 0.68)% vs (5.47 ± 0.95)%, *P* > 0.05] before irradiation. However, a significant difference was found between CNE2R-CD166(+) and CNE2R-CD166(−) cells [(16.13 ± 1.82)% vs (31.57 ± 0.95)%, *P* > 0.05] after 10 Gy irradiation for 24 h.

## DISCUSSION

The radiotherapy technology for treating NPC has improved in recent years. However, still more than 15% of patients have NPC recurrence [8]. Radioresistance is one of the main factors in the current clinical management of NPC [4, 5]. A number of studies have been conducted on resistance-related factors, such as overexpression of DNA repair proteins [9], abnormal expression of epidermal growth factor receptor (EGFR) [10], autophagy [11–13], angiogenesis [14], and cancer stem cells [15]. However, no effective serum biomarkers are available at present for predicting the radiosensitivity of NPC. The proteomic technology is an effective method to search for effective serum biomarkers. In previous studies [7], some differentially secreted proteins were identified by proteomic techniques. CD166 is one of the 26 differentially secreted proteins, which is of interest.

CD166, also known as ALCAM, is a highly conserved 110-kDa multidomain transmembrane type-1 glycoprotein belonging to the immunoglobulin superfamily, which was first described as a CD6 ligand on leukocytes. It plays an important role in some biological activities, including proliferation, T-cell activation, and angiogenesis [16]. Through homophilic and heterophilic adhesion, CD166 mediates the interaction between cells and participates in many kinds of pathological and physiological processes [17]. It has been confirmed that CD166 is abnormally expressed in a variety of tumor cells and plays an important role in tumor invasion and metastasis, such as breast cancer [18], liver cancer [19], stomach cancer [20], colon cancer [21], malignant melanoma [22], salivary gland tumor [23], prostate cancer [24], and so forth. It is also thought to be one of the markers of cancer stem cells [25]. A study [26] showed that the expression rate of CD166 in head and neck squamous cell carcinomas (HNSCCs, including oral cavity, oropharynx, and hypopharynx/larynx) was up to 70.32%, and CD166 expression was significantly more frequent in malignant tissues compared with nonmalignant tissues. Another study of HNSCCs [27] demonstrated that CD166 was a valuable cell surface marker for the enrichment of HNSCC stem cells and the level of CD166 expression was associated with the tumor recurrence rate. However, the two aforementioned studies did not include NPC. In 1976 [28], the cancer stem cell theory was first proposed by Nowell: stem cells are rare in most tissues, and cancer is thought to be a kind of disease of stem cells [29]. Furthermore, the existence of cancer stem cells is one of the causes of resistance [15]. However, in this study, the positive rate of CD166 in CNE2 was (21.37 ± 1.50)%, which did not support the hypothesis that it is the stem cell marker of NPC.

Moreover, CD166 and EGFR are involved in protein–protein interactions [30]. EGFR, a key member of the tyrosine kinase receptor family, is a transmembrane tyrosine kinase protein of 170 kDa encoded by EGFR gene. It is involved in the occurrence and development of malignant tumors by regulating the cell cycle, apoptosis, and so on [31]. EGFR can significantly promote cell proliferation [32]. EGFR gene silencing can inhibit the growth of NPC cells, increase cell apoptosis, and increase the sensitivity of cells to chemotherapy [33]. The overexpression of EGFR was also associated with a poor effect of tumor radiotherapy [34]. In head and neck cancer cells, high levels of EGFR are associated with radiation resistance, whereas the anti-EGFR monoclonal antibody can improve the radiosensitivity of cells [35, 36]. In advanced or recurrent NPC, the expression of EGFR has a direct effect on the radiosensitivity of cells. Patients whose tumors expressed low levels of CD166 performance were more sensitive to radiation compared with those whose tumors expressed low high levels of CD166 [37]. The Ras/Raf/MEK/MAPK and PI3K/AKT/mTOR pathways have been well studied among the downstream signaling pathways activated by EGFR. CD166 may play an important role in antiradiation activity through the EGFR signaling pathway. Therefore, the gene silencing of CD166 in NPC cells may be one of the effective strategies to enhance the radioresistance of cells and is worth further exploration.

**Figure 5:**
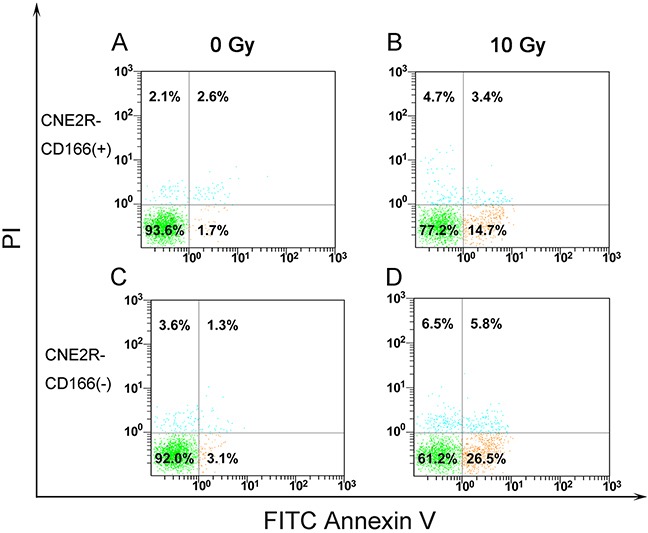
**(A) Apoptotic rates of CNE2R-CD166(+) cells before irradiation. (B)** Apoptotic rates of CNE2R-CD166(+) cells after 10 Gy irradiation. **(C)** Apoptotic rates of CNE2R-CD166(−) cells before irradiation. **(D)** Apoptotic rates of CNE2R-CD166(−) cells after 10 Gy irradiation.

The concentration of secreted protein CD166 was detected by ELISA in the present study. The concentration of secreted protein CD166 was significantly higher in the radioresistant group than in the radiosensitive group (Figure 1). No correlation was found between CD166 expression and clinical stage, T stage, N stage, and other clinical parameters, but it was not due to the lack of clinical samples. Next, the flow cytometric analysis showed that the CD166 positive rate in the CNE2 cell membrane was significantly higher than that in the CNE2R cell membrane (Figure 2). CNE2R-CD166(+) and CNE2R-CD166(−) cells were obtained according to the MACS technology for the following experiments. Radiosensitivity, cell proliferation, and apoptosis were assessed using colony formation assay, CCK-8 assay, and flow cytometry, respectively. The result of colony formation assay indicated that CNE2R-CD166(+) cells were more radioresistant compared with CNE2R-CD166(−) cells (Figure 3 and Table 2). The result of CCK-8 assay indicated that the survival fraction of CNE2R-CD166(+) cells was significantly higher than that of CNE2R-CD166(−) cells (Figure 4 and Table 3). The main mechanism of tumor radiotherapy was to induce cell apoptosis [38]. The apoptotic rates of CNE2R-CD166(+) cells after irradiation was significantly lower than that of CNE2R-CD166(−) cells in this study (Figure 5).

**Table 3:** Comparison of clinical–pathological parameters in the two groups of nasopharyngeal carcinoma

Classification		Radioresistant group(*n* = 30)	Radiosensitive group(*n* = 30)	*p
Pathologic type	Differentiated	5	6	>0.05
	Undifferentiated	25	24	
T Stage	T1	2	3	>0.05
	T2	4	10	
	T3	9	6	
	T4	15	11	
N Stage	N0	0	0	>0.05
	N1	15	10	
	N2	13	16	
	N3	2	4	
Clinical stage	I	0	0	>0.05
	II	3	6	
	III	11	9	
	IVa+IVb	16	15	
Sex	Male	20	25	>0.05
	Female	10	5	
Age	<50	17	16	>0.05
	≥50	13	14	
Hemoglobin (g/L)		132.6 ± 16.89	131.50 ± 16.30	>0.05
GTVnx (Gy)		71.76 ± 0.92	71.52 ± 1.05	>0.05
GTVnd (Gy)		68.34 ± 1.80	68.25 ± 2.69	>0.05
CTV1 (Gy)		60.73 ± 0.98	60.33 ± 0.78	>0.05
CTV2 (Gy)		54.66 ± 0.88	54.23 ± 0.80	>0.05

* The difference between the two groups was not statistically significant.

In conclusion, CD166 is a novel biomarker for predicting NPC response to radiotherapy. The individual treatment should be planned according to the expression of CD166 in the serum of NPC before treatment.

## MATERIALS AND METHODS

### Serum sample

A total of 60 NPC serum samples obtained from the Affiliated Cancer Hospital of Guangxi Medical University from March 2015 to October 2013 were used in this study. All the patients were diagnosed by histopathology, and TNM stage designation was according to the definitions of the seventh edition of the Union of International Cancer Control American Joint Committee on Cancer staging criteria. All the patients had no distant metastasis and did not receive radiotherapy or chemotherapy before the acquisition of serum. The patients were treated with 6 MV X-radiation from an Elekta linear accelerator (Precise 1120; Elekta Instrument AB, Stockholm, Sweden) at a dose rate of 220 cGy/min. After 7–8 weeks of radiation therapy, the patients were divided into the radiosensitive group and the radioresistant group based on the therapeutic effect [39]. Patients with radioresistant NPC were defined as the ones with persistent disease (incomplete regression of primary tumor and/or neck lymph nodes) at >3 months or with the local recurrent disease at the nasopharynx and/or neck lymph nodes at ≤12 months after completion of radiotherapy. Patients with radiosensitive NPC were defined as the ones without the local residual lesions (complete regression) at >3 months and without local recurrent disease at >12 months after completion of radiotherapy. Each group included 30 patients, and the difference between the 2 groups was not statistically significant. The detailed clinical data are shown in Table 3.

### Enzyme-linked immunosorbent assay for the determination of serum C166 concentration

CD166 concentrations in the serum of patients with NPC were quantified using an ALCAM (human) Enzyme-linked Immunosorbent Assay (ELISA) Kit (Catalog Number KA1692, Abnova). All reagents, samples, and standards were prepared as instructed. All standards and samples were run in duplicate and the dilution for serum is 200 fold. Add 100 μL standard or sample to each well. Incubate 2.5 hours at room temperature. Discard the solution and wash 4 times with 1x Wash Solution. Add 100 μL prepared biotin antibody to each well and Incubate 1 hour at room temperature. Repeat the wash steps, then add 100 μL prepared Streptavidin solution and incubate 45 minutes at room temperature. Repeat the wash steps again. Add 100 μL TMB One-Step Substrate Reagent to each well and incubate 30 minutes at room temperature. Add 50 μL Stop Solution to each well. Read at a microplate reader (Bio-Rad, Hercules, CA, USA) at 450 nm immediately. The mean absorbance for each set of duplicate standards, controls, and samples was calculated. The standard curve was plotted using SigmaPlot software to calculate the CD166 concentration of each sample.

### Cell lines and culture

CNE-2, a human NPC cell line, was constructed by the Cancer Hospital Chinese Academy of Medical Science and Guangdong Medical University and purchased from the Cancer Hospital of Shanghai Fudan University. The radioresistant cell line CNE2R was obtained from CNE2 after exposure to radiation at a total dose of 64 Gy in 1 year by the Research Department, Affiliated Cancer Hospital of Guangxi Medical University [40]. CNE-2 and CNE-2R cells were cultured in RPMI-1640 medium (Hyclone, USA) with the addition of 10% fetal bovine serum(FBS) (Gibco, USA), penicillin (100 U/ml) and streptomycin (100 μg/ml), and were cultured in a humidified 5% CO_2_ atmosphere at 37°C.

### Positive rate of CD166 in the cell membrane was detected by flow cytometry, and the MACS technology was used to sort cells

CNE2 and CNE2R cells were cultured as previously until the cells grew to 80% confluence. The cells were digested with 0.25% trypsin-0.02% EDTA. They were washed twice with phosphate-buffered saline (PBS) to remove any adherent serum, and 5 × 10^6^ cells were resuspended in 100 μL of PBS. The cells were labeled with 10 μL of phycoerythrin (PE)-conjugated anti-CD166 antibody (BD Pharmingen, CA, USA), incubated for 10 min in the dark in the refrigerator (2°C−8°C), and detected using a FC500 flow cytometry systems(Beckman Coulter). Isotype-matched mouse antibodies served as controls. The experiments were performed three times.

NEXT, We use MiniMASC^TM^ Starting Kit (Miltenyi Biotec, Germany) for cell sorting. When incubate for 10 minutes in the dark in the refrigerator (2−8°C) as previously, wash cells to remove unbound primary antibody. Aspirate supernatant completely and resuspend cell pellet in 80 μL of buffer per 10^7^ total cells. Add 20 μL of Anti-PE MicroBeads per 10^7^ total cells. Mix well and incubate for 15 minutes in the refrigerator(2−8°C). Wash cells by adding 2mL of buffer per 10^7^ cells and centrifuge at 300×g for 10 min. Resuspend up to 10^8^ total cells in 500μl of buffer. After magnetic labeling, cells are passed through a MACS Column which is placed in the strong permanent magnet of the MACS Separator. Unlabeled cells passed through while magnetically labebled cell are retained in the column. Remove column from the separator and place it on a suitable collection tube, then pipette appropriate amount of buffer onto the column. Immediately flush out the magnetically labeled cells by firmly pushing the plunger into the column.

In order to verify the purity of the separation, after separation the PE-labeled cells were detected by flow cytometry directly. Then CNE2R-CD166 (+), CNE2R-CD166 (−) cells were cultured for further experiments.

### Colony formation assay

The colony formation assay is the standard to detect the sensitivity of cells to radiation [41]. Aliquots consisting of 200, 200, 400, 600 and 1,000 CNE2R-CD166(+) and CNE2R-CD166(−) cells were plated into five 6-well plates and individually exposed to doses of 0, 2, 4, 6, and 8 Gy with 6 MV X-rays from an Elekta linear accelerator (Precise 1120; Elekta Instrument AB, Stockholm, Sweden) with a dose rate of 220 cGy/min. Culture solution was replaced every 2-3 days after irradiation until twelfth days. Then the colonies were fixed with carbinol for 20 min and stained with 0.1% Giemsa (Solarbio, Pe King) for 30 min. Colonies (defined as any colony with ≥50 cells) were scored as survivors. All experiments were performed three times. The dose responses were analyzed using the multitarget single-hit model, SF = 1 – (1 – e^−*D*/*D*0^)*^N^*, where D is the single radiation dose, D_0_ is the single radiation dose of radiation producing a 37% survival rate, SF is the survival fraction at dose D and N is the radiobiological parameter.

### CCK-8 assay

Cell viability in response to irradiation was detected by the CCK-8 assay (Dojindo, Japan). Cells were plated in four 96-well plates at a density of 4×10^3^ cells/well and allowed to attach for 24h. The cells were then irradiated with 6 MV X-radiation at a dose of 10 Gy. After culturing for 0, 24, 48, and 72 h, 10 μL of 10 mg/mL CCK-8 solution was added to each well and cultured in a humidified 5% CO_2_ atmosphere at 37°C for 2 h. The absorbance of each well was measured using a microplate reader at 450 nm. Five wells for each time point, all experiments were performed three times. OD is the mean absorbance after remove a maximum and a minimum OD values. Cell survival fraction was calculated using the formula:Survival fraction (%)= OD/OD_0h_ ×100%.

### Apoptosis analysis

CNE-2R-CD166(+) and CNE-2R-CD166(−) cells were seeded in 6-well culture plates at 5 × 10^5^ cells/well and allowed to attach for 24 h. Then, the cells were irradiated with 6 MV X-radiation at a dose of 10 Gy. After culturing in a humidified 5% CO_2_ atmosphere at 37°C for 24 h, the cells were collected and resuspended in PBS and stained first with Annexin-V–fluorescein isothiocyanate (FITC) for 10 min at room temperature and then with propidium iodide for 10 min in the dark, according to the manufacturer's instructions of the FITC Annexin V apoptosis detection kit I (BD Pharmingen, USA). The cells were analyzed immediately on the FC500 flow cytometry systems. All samples were assayed in triplicate.

### Statistical analysis

Data were presented as the mean ± standard deviation(SD). Statistical analyses were performed using SPSS 17.0 (SPSS, IL, USA) or GraphPad Prism 5.0 software. The Wilcoxon rank-sum test was used to analyze the CD166 expression in different groups. The independent-samples *t* test was used to analyze the cell proliferation and apoptotic rates. All statistical tests were two sided. A *p* value less than 0.05 indicated statistically significant differences.
